# Safety and efficacy of cabozantinib for patients with advanced hepatocellular carcinoma who advanced to Child–Pugh B liver function at study week 8: a retrospective analysis of the CELESTIAL randomised controlled trial

**DOI:** 10.1186/s12885-022-09453-z

**Published:** 2022-04-09

**Authors:** Anthony B. El-Khoueiry, Tim Meyer, Ann-Lii Cheng, Lorenza Rimassa, Suvajit Sen, Steven Milwee, Robin Kate Kelley, Ghassan K. Abou-Alfa

**Affiliations:** 1 USC Norris Comprehensive Cancer Center, Los Angeles, CA USA; 2 Royal Free Hospital, University College London, London, UK; 3 National Taiwan University Hospital, Taipei, Taiwan; 4 Department of Biomedical Sciences, Humanitas University, Pieve Emanuele, Milan, Italy; 5 Medical Oncology and Hematology Unit, Humanitas Cancer Center, IRCCS Humanitas Research Hospital, Rozzano, Milan Italy; 6 Exelixis, Inc, Alameda, CA USA; 7 UCSF Helen Diller Family Comprehensive Cancer Center, CA San Francisco, USA; 8 Memorial Sloan Kettering Cancer Center, New York, NY USA; 9 Weill Medical College at Cornell University, New York, NY USA

**Keywords:** Cabozantinib, Child–Pugh B, Hepatocellular carcinoma

## Abstract

**Background:**

Patients with hepatocellular carcinoma (HCC) and Child–Pugh B liver cirrhosis have poor prognosis and are underrepresented in clinical trials. The CELESTIAL trial, in which cabozantinib improved overall survival (OS) and progression-free survival (PFS) versus placebo in patients with HCC and Child–Pugh A liver cirrhosis at baseline, was evaluated for outcomes in patients who had Child–Pugh B cirrhosis at Week 8.

**Methods:**

This was a retrospective analysis of adult patients with previously treated advanced HCC. Child–Pugh B status was assessed by the investigator. Patients were randomised 2:1 to cabozantinib (60 mg once daily) or placebo.

**Results:**

Fifty-one patients receiving cabozantinib and 22 receiving placebo had Child–Pugh B cirrhosis at Week 8. Safety and tolerability of cabozantinib for the Child–Pugh B subgroup were consistent with the overall population. For cabozantinib- versus placebo-treated patients, median OS from randomisation was 8.5 versus 3.8 months (HR 0.32, 95% CI 0.18–0.58), median PFS was 3.7 versus 1.9 months (HR 0.44, 95% CI 0.25–0.76), and best response was stable disease in 57% versus 23% of patients.

**Conclusions:**

These encouraging results with cabozantinib support the initiation of prospective studies in patients with advanced HCC and Child–Pugh B liver function.

Clinical Trial Registration: NCT01908426.

**Supplementary Information:**

The online version contains supplementary material available at 10.1186/s12885-022-09453-z.

## Background

Most patients with advanced hepatocellular carcinoma (HCC) present with underlying cirrhosis, the severity of which can be indicated using Child–Pugh assessments [1–5]. The majority of systemic therapies for advanced HCC have been studied in large prospective randomised studies in the Child–Pugh A population, as most of these trials excluded patients with poor liver function (Child–Pugh B or worse hepatic dysfunction). Further, underlying liver cirrhosis represents a competing risk of death in patients with HCC and Child–Pugh B cirrhosis; therefore, the benefit of anticancer therapy is difficult to evaluate in non-randomised studies. Consequently, limited data are available for the use of systemic therapies in patients with advanced liver cirrhosis, resulting in a lack of treatment options for this population [6–8].

Cabozantinib is a tyrosine kinase inhibitor with targets that include MET, VEGFR, and the TAM family of receptor kinases and is approved for patients with HCC who have been previously treated with sorafenib [9, 10]. In the pivotal phase 3 CELESTIAL trial (NCT01908426), cabozantinib, as second- or third-line therapy, significantly improved overall survival (OS) and progression-free survival (PFS) versus placebo in patients with previously treated advanced HCC and Child–Pugh A liver cirrhosis [11]. Median OS was 10.2 months with cabozantinib versus 8.0 months with placebo (hazard ratio [HR] 0.76; 95% confidence interval [CI] 0.63–0.92; *p* = 0.005), and median PFS was 5.2 months with cabozantinib versus 1.9 months with placebo (HR 0.44; 95% CI 0.36–0.52; *p* < 0.001) [11].

We present a post hoc retrospective evaluation of the safety and efficacy of cabozantinib in patients from CELESTIAL with Child–Pugh A liver cirrhosis who progressed to Child–Pugh B cirrhosis at Week 8. The objective of this analysis was to characterise clinical outcomes in this cohort of patients.

## Methods

This is a retrospective analysis of outcomes from CELESTIAL for the subgroup of patients who had Child–Pugh B cirrhosis, as assessed by the investigator, by Week 8 (time of first Child–Pugh assessment and the first radiographic assessment after randomisation). Child–Pugh scoring was also independently determined retrospectively by the Biostatistics and Clinical Data Management (BCDM) department at Exelixis Inc. (study sponsor), based on investigator assessments for ascites and hepatic encephalopathy and central laboratory assessments. CELESTIAL study details have been previously published for the efficacy and safety results for the overall population [11]. The study allowed adult patients with advanced HCC, Child–Pugh class A liver function, and Eastern Cooperative Oncology Group performance status of 0 or 1 [11]. Patients must have received prior sorafenib and could have received up to two prior systemic regimens [11]. Patients were randomised 2:1 to receive cabozantinib 60 mg once daily or matched placebo [11]. Randomisation was performed with an interactive response system and permuted blocks. Randomisation was stratified by disease aetiology (hepatitis B virus [HBV], with or without hepatitis C virus [HCV]; HCV without HBV; or non-viral), geographic region (Asia or other), and extrahepatic spread of disease, macrovascular invasion, or both (yes or no). The outcomes reported in this retrospective analysis are safety, with assessments starting from study initiation; OS; and investigator-assessed PFS and tumour response per RECIST v1.1. Overall survival was defined as the time from randomisation to death from any cause; progression-free survival was defined as the time from randomisation to radiographic progression or death from any cause, whichever occurred first; objective response rate was defined as the percentage of patients with a confirmed complete or partial response. Adverse events (AEs) were reported according to National Cancer Institute Common Terminology Criteria for Adverse Events v4.0 [12]. Radiographic assessment by computed tomography or magnetic resonance imaging was conducted every 8 weeks after randomisation with a follow-up assessment conducted 8 weeks after radiographic progression or treatment discontinuation. Safety was assessed continuously with a final assessment 30 days after treatment discontinuation. Patients who were Child–Pugh A at Week 8 or patients who did not have a Child–Pugh assessment at Week 8 were excluded from this retrospective analysis; patients with Child–Pugh C status at Week 8 were also excluded as cabozantinib should be avoided in patients with severe hepatic impairment. The data cutoff date was 1 June 2017.

## Results

### Patient Population

At randomisation, nearly all patients had investigator-assessed Child–Pugh class A cirrhosis, with seven patients in the cabozantinib arm and two patients in the placebo arm assessed to have Child–Pugh B cirrhosis and noted as protocol deviations. Three out of a total of nine patients with Child–Pugh B status at baseline had Week 8 data, with all being in the cabozantinib group; one remained with Child–Pugh B and two were assessed with Child–Pugh A at Week 8. At the time of the first Child–Pugh assessment at Week 8 after randomisation, 51/470 patients in the cabozantinib arm and 22/237 patients in the placebo arm had investigator-assessed Child–Pugh B cirrhosis (Child–Pugh B subgroup). Child–Pugh status at Week 8 was unknown for 288 patients in the overall study population (194 for cabozantinib and 94 for placebo), Child–Pugh A for 343 patients (223 and 120), and Child–Pugh C for 3 patients (2 and 1); these patients were excluded from this retrospective analysis. Cabozantinib and placebo were received for ≥ 8 weeks by 94% (48/51) and 82% (18/22) of patients, respectively, for the Child–Pugh B cohort and 80% (375/467) and 76% (135/237) of patients, respectively, for the overall population. As of data cutoff, the percent (n) of patients who were still on cabozantinib/placebo was 6% (3)/0 for the Child–Pugh B cohort and 16% (73)/11% (26) for the overall population.

For patients with investigator-assessed Child–Pugh B status at Week 8, the majority (64%) had a BCDM-determined Child–Pugh score of A6 at baseline and 27% had a Child–Pugh score of A5, whereas 7% had a score ≥ 7 and 1% of scores were missing (Table 1). Among those who still had investigator-assessed Child–Pugh A cirrhosis at Week 8, 26% (90/341) had BCDM-determined Child–Pugh A6 status and 72% (246/341) had A5 status at baseline. In the overall CELESTIAL patient population, 37% had Child–Pugh A6 status and 59% had Child–Pugh A5 status at baseline. At least half of the patients (51%) in the Child–Pugh B subgroup had a Child–Pugh score of 7 at Week 8, whereas 19% and 11% had Child–Pugh scores of 8 and 9, respectively (Table 2). Point changes from baseline in the levels of albumin, bilirubin, and ascites were the most common contributors to the development of the Child–Pugh B status at Week 8 for patients in both the cabozantinib and placebo arms. At Week 8, greater changes from baseline were observed for the Child–Pugh B subgroup versus the overall population in various liver function parameters, including liver enzyme activity (i.e., alkaline phosphatase), and albumin and bilirubin levels (Additional file 1, Table 1).

**Table 1:** Baseline characteristics of Child–Pugh B subgroup

	**Child–Pugh B subgroup**			**Overall population**^a^		
	**Cabozantinib** **(*N* = 51)**	**Placebo** **(*N* = 22)**	**Total** **(*N* = 73)**	**Cabozantinib** (***N* = 470)**	**Placebo** **(*N* = 237)**	**Total** **(*N* = 707)**
Median age (range), years	63.0 (22–82)	64.5 (50–85)	64.0 (22–85)	64 (22–86)	64 (24–86)	64 (22–86)
Male, n (%)	45 (88)	20 (91)	65 (89)	379 (81)	202 (85)	581 (82)
Geographic region, n (%)						
Asia	14 (27)	3 (14)	17 (23)	116 (25)	59 (25)	175 (25)
Europe	21 (41)	12 (55)	33 (45)	231 (49)	108 (46)	339 (48)
Australian/New Zealand	1 (2)	1 (5)	2 (3)	15 (3)	11 (5)	26 (4)
Canada/USA	15 (29)	6 (27)	21 (29)	108 (23)	59 (25)	167 (24)
Race, n (%)						
Asian	17 (33)	5 (23)	22 (30)	159 (34)	82 (35)	241 (34)
White	30 (59)	14 (64)	44 (60)	264 (56)	130 (55)	394 (56)
Black	0	2 (9)	2 (3)	8 (2)	11 (5)	19 (3)
Other	1 (2)	1 (5)	2 (3)	8 (2)	2 (1)	10 (1)
Not reported	3 (6)	0	3 (4)	31 (7)	12 (5)	43 (6)
ECOG status, n (%)						
0	27 (53)	12 (55)	39 (53)	245 (52)	131 (55)	376 (53)
1	24 (47)	10 (45)	34 (47)	224 (48)	106 (45)	330 (47)
2	0	0	0	1 (< 1)	0	1 (< 1)
Aetiology of disease, n (%)						
HBV	18 (35)	6 (27)	24 (33)	178 (38)	89 (38)	267 (38)
HCV	16 (31)	4 (18)	20 (27)	113 (24)	55 (23)	168 (24)
Alcohol use	19 (37)	4 (18)	23 (32)	112 (24)	39 (16)	151 (21)
Nonalcoholic steatohepatitis	3 (6)	2 (9)	5 (7)	43 (9)	23 (10)	66 (9)
AFP, n (%)						
< 400 ng/mL	31 (61)	16 (73)	47 (64)	278 (59)	136 (57)	414 (59)
≥ 400 ng/mL	20 (39)	6 (27)	26 (36)	192 (41)	101 (43)	293 (41)
Albumin, n (%)						
< 35 g/L	27 (53)	11 (50)	38 (52)	131 (28)	60 (25)	191 (27)
≥ 35 g/L	24 (47)	11 (50)	35 (48)	339 (72)	177 (75)	516 (73)
Bilirubin, n (%)						
< 22.23 µmol/L	40 (78)	20 (91)	60 (82)	421 (90)	221 (93)	642 (91)
≥ 22.23– < 29.07 µmol/L	6 (12)	2 (9)	8 (11)	37 (8)	13 (5)	50 (7)
≥ 29.07 µmol/L	5 (10)	0	5 (7)	12 (3)	3 (1)	15 (2)
Extrahepatic spread of disease and/or macrovascular invasion, n (%)	47 (92)	17 (77)	64 (88)	398 (85)	200 (84)	598 (85)
Extrahepatic spread of disease	42 (82)	15 (68)	57 (78)	369 (79)	182 (77)	551 (78)
Macrovascular invasion	22 (43)	7 (32)	29 (40)	129 (27)	81 (34)	210 (30)
ALBI grade, n (%)						
1	5 (10)	1 (5)	6 (8)	186 (40)	102 (43)	288 (41)
2	45 (88)	21 (95)	66 (90)	282 (60)	133 (56)	415 (59)
3	1 (2)	0	1 (1)	2 (< 1)	2 (1)	4 (1)
Child–Pugh score, n (%)^b^						
5	13 (25)	7 (32)	20 (27)	264 (56)	153 (65)	417 (59)
6	33 (65)	14 (64)	47 (64)	183 (39)	78 (33)	261 (37)
≥ 7	4 (8)	1 (5)	5 (7)	17 (4)	5 (2)	22 (3)
Missing	1 (2)	0	1 (1)	6 (1)	1 (< 1)	7 (1)
Sites of disease, n (%)						
Liver	45 (88)	21 (95)	66 (90)	395 (84)	216 (91)	611 (86)
Bone	9 (18)	2 (9)	11 (15)	60 (13)	34 (14)	94 (13)
Visceral (excluding liver)	24 (47)	9 (41)	33 (45)	215 (46)	105 (44)	320 (45)
Lymph node	19 (37)	3 (14)	22 (30)	155 (33)	71 (30)	226 (32)
Number of prior systemic anticancer regimens for advanced HCC, n (%)						
0	1 (2)	0	1 (1)	3 (1)	0	3 (< 1)
1	33 (65)	13 (59)	46 (63)	335 (71)	174 (73)	509 (72)
2	16 (31)	9 (41)	25 (34)	130 (28)	62 (26)	192 (27)
≥ 3	1 (2)	0	1 (1)	2 (< 1)	1 (< 1)	3 (< 1)
TACE for HCC, N (%)	26 (51)	13 (59)	39 (53)	203 (43)	111 (47)	314 (44)
Median total duration of prior sorafenib (range), months	5.4 (1.1–40.0)	7.1 (1.0–29.2)	5.4 (1.0–40.0)	5.3 (0.3–70.0)	4.8 (0.2–76.8)	5.2 (0.2–76.8)
Median time from disease progression to randomisation (range), mo^c^	1.5 (0.2–100.8)	1.9 (0.4–69.4)	1.5 (0.2–100.8)	1.6 (0–100.8)	1.7 (0.2–69.4)	1.6 (0–100.8)

^a^Data from Abou-Alfa et al. N. Engl. J. Med. 379, 54–63 (2018) [11]. ^b^As Child–Pugh grading was investigator assessed and Child–Pugh scoring was determined retrospectively by BCDM, some discrepancies between grading and scoring results existed. ^c^*n* = 49 and 21 for cabozantinib and placebo cohorts, respectively. *AFP* alpha fetoprotein, *ALBI* albumin-bilirubin, *BCDM* Biostatistics and Clinical Data Management, *ECOG* Eastern Cooperative Oncology Group, *HBV* hepatitis B virus, *HCV* hepatitis C virus, *TACE* transarterial chemoembolisation

**Table 2:** Child–Pugh scores at Week 8

	**Patients with Child–Pugh B at Week 8,* n***	**Patients with available BCDM-determined Child–Pugh score points, *n ***^**a**^	**Child–Pugh score (Week 8)** ***n* (%)** ^**b**^		
			**7 points**	**8 points**	**9 points**
Cabozantinib	51	42	26 (51)	11 (22)	3 (6)
Placebo	22	21	11 (50)	3 (14)	5 (23)

^a^Two patients each in the cabozantinib and placebo cohorts had a score of 6. As Child–Pugh grading was investigator assessed and Child–Pugh scoring was determined independently by BCDM, some discrepancies between grading and scoring results existed. ^b^Percentage of total number of patients who developed Child–Pugh B cirrhosis. *BCDM* Biostatistics and Clinical Data Management

Patients in the Child–Pugh B subgroup tended to have higher baseline rates of albumin-bilirubin (ALBI) grades 2/3 compared with the overall study population (92% vs. 59%), macrovascular invasion (40% vs. 30%), and prior transarterial chemoembolisation for HCC (53% vs. 44%), whereas aetiology of hepatitis B virus (HBV) tended to be lower (33% vs. 38%) (Table 1). In the Child–Pugh B subgroup, patients in the cabozantinib arm versus the placebo arm tended to have higher baseline rates of macrovascular invasion (43% vs. 32%), extrahepatic spread (82% vs. 68%), alpha-fetoprotein ≥ 400 ng/mL (39% vs. 27%), HBV (35% vs. 27%), and hepatitis C virus (31% vs. 18%). Additionally, for the Child–Pugh B subgroup, the cabozantinib arm in comparison with the placebo arm tended to have a higher baseline rate of ALBI grade 1 (10% vs. 5%) and a lower rate of ALBI grade 2 (88% vs. 95%).

### Safety and Tolerability

For patients assigned to cabozantinib, the median average daily dose (36.9 mg), the median duration of exposure (3.7 months), and the rates of dose reduction (61%) and discontinuation (18%) due to treatment-related AEs for patients in the Child–Pugh B subgroup were similar to the overall cabozantinib group (Table 3). Grade 3/4 all-causality AEs in the cabozantinib arm were experienced by 71% of patients in the Child–Pugh B subgroup compared with 68% overall. The rates of the most common grade 3/4 AEs were numerically higher in the Child–Pugh B subgroup compared with the overall cabozantinib group for fatigue (20% vs. 10%), ascites (14% vs. 4%), and thrombocytopenia (12% vs. 3%) and lower for palmar-plantar erythrodysesthesia (8% vs. 17%) and hypertension (8% vs. 16%). Rates of grade 3/4 AEs associated with liver toxicity were generally similar for the Child–Pugh B subgroup compared with the overall cabozantinib group, with rates comparable for increased alanine aminotransferase (ALT) and aspartate aminotransferase (AST) and higher for increased bilirubin (10% vs. 3%). The occurrence of grade 3/4 AEs associated with cirrhosis decompensation was greater for the Child–Pugh B subgroup than the overall cabozantinib group for ascites, indicated previously, and hepatic encephalopathy (6% vs. 3%).

**Table 3:** Safety and tolerability of cabozantinib (safety population)

	**Child–Pugh B subgroup**				**Overall population**^**a**^			
	**Cabozantinib (*N* = 51)**		**Placebo (*N* = 22)**		**Cabozantinib (*N* = 467)**		**Placebo (*N* = 237)**	
Median duration of exposure (range), months	3.7 (1.4–12.9)		2.0 (0.9–5.5)		3.8 (0.1–37.3)		2.0 (0.0–27.2)	
Median average daily dose (range), mg	36.9 (12.5–60.0)		56.8 (17.9–60.0)		35.8 (1.1–60.0)		58.9 (12.0–60.0)	
Dose reduction, n (%)	31 (61)		3 (14)		291 (62)		30 (13)	
Discontinuation due to treatment-related AE, n (%)	9 (18)		1 (5)		74 (16)		6 (2.5)	
All-causality AE, n (%)^b^	Any grade	Grade 3/4	Any grade	Grade 3/4	Any grade	Grade 3/4	Any grade	Grade 3/4
Any event	51 (100)	36 (71)	22 (100)	13 (59)	460 (99)	316 (68)	219 (92)	86 (36)
Fatigue	29 (57)	10 (20)	9 (41)	4 (18)	212 (45)	49 (10)	70 (30)	10 (4.2)
Ascites	17 (33)	7 (14)	12 (55)	5 (23)	57 (12)	18 (3.9)	30 (13)	11 (4.6)
AST increased	11 (22)	7 (14)	2 (9.1)	1 (4.5)	105 (22)	55 (12)	27 (11)	16 (6.8)
Thrombocytopenia	11 (22)	6 (12)	0	0	52 (11)	16 (3.4)	1 (0.4)	0
Anaemia	6 (12)	5 (9.8)	5 (23)	4 (18)	46 (9.9)	19 (4.1)	19 (8.0)	12 (5.1)
Blood bilirubin increased	11 (22)	5 (9.8)	3 (14)	0	45 (9.6)	14 (3.0)	17 (7.2)	4 (1.7)
Dyspnoea	10 (20)	5 (9.8)	7 (32)	0	58 (12)	15 (3.2)	24 (10)	1 (0.4)
Blood ALP increased	4 (7.8)	4 (7.8)	0	0	34 (7.3)	16 (3.4)	14 (5.9)	1 (0.4)
Hypertension	9 (18)	4 (7.8)	0	0	137 (29)	74 (16)	14 (5.9)	4 (1.7)
PPE	15 (29)	4 (7.8)	1 (4.5)	0	217 (46)	79 (17)	12 (5.1)	0
Platelet count decreased	6 (12)	4 (7.8)	0	0	45 (9.6)	17 (3.6)	7 (3.0)	2 (0.8)
Portal vein thrombosis	4 (7.8)	4 (7.8)	0	0	6 (1.3)	5 (1.1)	0	0
Pulmonary embolism	4 (7.8)	4 (7.8)	0	0	7 (1.5)	6 (1.3)	5 (2.1)	4 (1.7)
Asthenia	12 (24)	3 (5.9)	3 (14)	0	102 (22)	32 (6.9)	18 (7.6)	4 (1.7)
Decreased appetite	30 (59)	3 (5.9)	5 (23)	0	225 (48)	27 (5.8)	43 (18)	1 (0.4)
Diarrhoea	24 (47)	3 (5.9)	6 (27)	1 (4.5)	251 (54)	46 (9.9)	44 (19)	4 (1.7)
General physical health deterioration	5 (9.8)	3 (5.9)	2 (9.1)	2 (9.1)	33 (7.1)	21 (4.5)	11 (4.6)	6 (2.5)
Hepatic encephalopathy	4 (7.8)	3 (5.9)	0	0	19 (4.1)	13 (2.8)	3 (1.3)	2 (0.8)
Hyperbilirubinemia	4 (7.8)	3 (5.9)	1 (4.5)	0	11 (2.4)	6 (1.3)	8 (3.4)	5 (2.1)
Nausea	23 (45)	3 (5.9)	6 (27)	0	147 (31)	10 (2.1)	42 (18)	4 (1.7)
Pain	3 (5.9)	3 (5.9)	0	0	19 (4.1)	4 (0.9)	5 (2.1)	0
Pneumonia	4 (7.8)	3 (5.9)	1 (4.5)	0	24 (5.1)	14 (3.0)	7 (3.0)	3 (1.3)
Abdominal pain	11 (22)	2 (3.9)	10 (45)	3 (14)	83 (18)	8 (1.7)	60 (25)	10 (4.2)
Hepatic failure	3 (5.9)	1 (2.0)	3 (14)	3 (14)	9 (1.9)	2 (0.4)	8 (3.4)	6 (2.5)
Sepsis	1 (2.0)	1 (2.0)	2 (9.1)	2 (9.1)	3 (0.6)	2 (0.4)	3 (1.3)	3 (1.3)
Additional events of interest								
ALT increased	7 (14)	2 (3.9)	1 (4.5)	0	80 (17)	23 (4.9)	13 (5.5)	5 (2.1)
Hyponatremia	5 (9.8)	2 (3.9)	0	0	26 (5.6)	18 (3.9)	9 (3.8)	5 (2.1)
Neutrophil count decreased	2 (3.9)	1 (2.0)	0	0	17 (3.6)	6 (1.3)	5 (2.1)	1 (0.4)
Hypoalbuminemia	17 (33)	1 (2.0)	2 (9.1)	0	55 (12)	2 (0.4)	12 (5.1)	0
Chronic hepatic failure	0	0	1 (4.5)	0	0	0	1 (0.4)	0

^a^Data from Abou-Alfa et al. N. Engl. J. Med. 379, 54–63 (2018) [11]. ^b^AEs of any cause that occurred at arate of > 5% for Grade 3/4 in either treatment arm of the Child–Pugh B subgroup or in the overall study population. Sorted by Grade 3/4 in the cabozantinib arm. Assessments starting from study initiation. *AE* adverse event, *ALT* alanine aminotransferase, *ALP* alkaline phosphatase, *AST* aspartate aminotransferase, *PPE* palmar-plantar erythrodysesthesia syndrome

For patients assigned to placebo, 59% in the Child–Pugh B subgroup and 36% overall experienced grade 3/4 all-causality AEs. Higher rates of Grade 3/4 AEs were reported in the Child–Pugh B subgroup relative to the overall placebo group for fatigue (18% vs. 4%) and ascites (23% vs. 5%), whereas rates were comparable for increased ALT, AST, and bilirubin.

### Efficacy Outcomes

**Figure 1:**
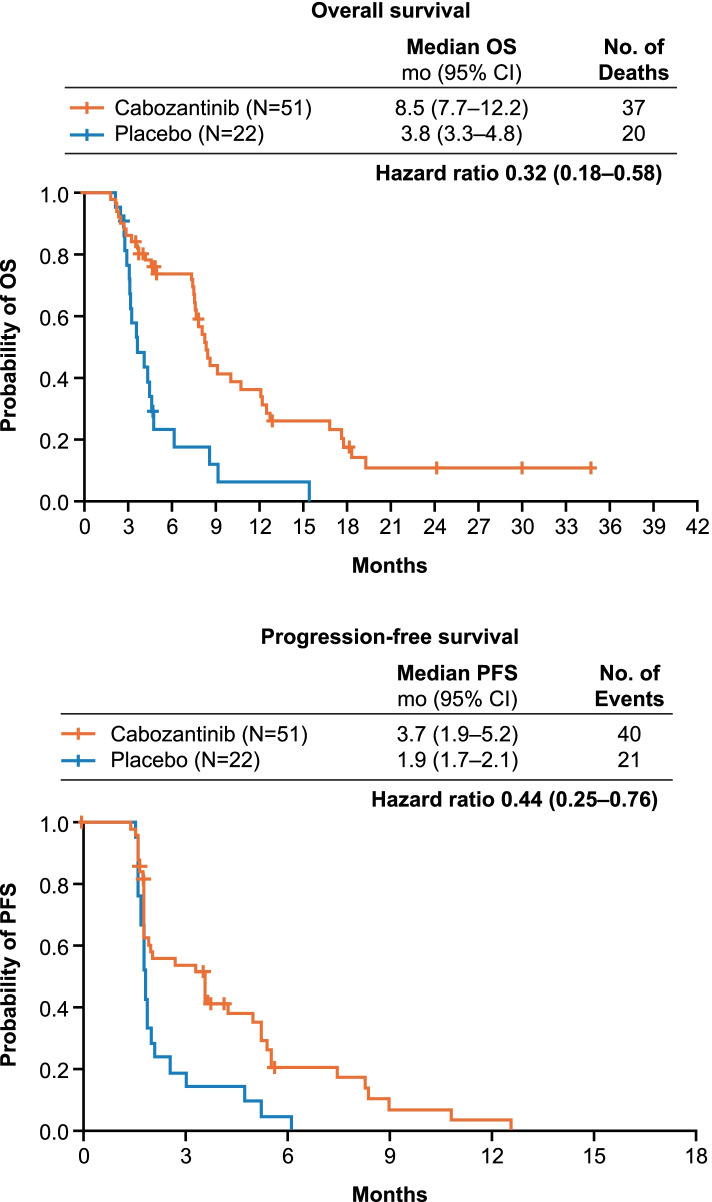
Fig 1 Overall survival and progression-free survival in the Child–Pugh B subgroup. *CI*, confidence interval; *mo*, months; *no*, number; *OS* overall survival, *PFS*, progression-free survival

In the Child–Pugh B subgroup, median OS was 8.5 months for patients receiving cabozantinib versus 3.8 months for patients receiving placebo (HR 0.32, 95% CI 0.18–0.58) (Fig. 1A). Median PFS was 3.7 months with cabozantinib versus 1.9 months with placebo (HR 0.44, 95% CI 0.25–0.76) (Fig. 1B). There were no complete or partial responses in the Child–Pugh B subgroup. Stable disease as a best objective response was obtained by 57% of patients in the cabozantinib arm versus 23% of patients in the placebo arm of the Child–Pugh B subgroup (Table 4). These results are consistent with those reported for the overall study population.

**Table 4:** Tumour response

	**Child–Pugh B subgroup**		**Overall population**^**a**^	
	**Cabozantinib (*N* = 51)**	**Placebo (*N* = 22)**	**Cabozantinib (*N* = 467)**	**Placebo (*N* = 237)**
Best overall response, n (%)				
Complete response	0	0	0	0
Partial response	0	0	18 (4)	1 (< 1)
Stable disease	29 (57)	5 (23)	282 (60)	78 (33)
Progressive disease	21 (41)	15 (68)	98 (21)	131 (55)
Not evaluable or missing	1 (2)	2 (9)	72 (15)	27 (11)

^a^Data from Abou-Alfa et al. N. Engl. J. Med. 379, 54–63 (2018) [11]

For the overall study population, median OS was 10.2 months with cabozantinib and 8.0 months with placebo (HR 0.76; 95% CI 0.63–0.92), whereas median PFS was 5.2 months with cabozantinib and 1.9 months with placebo (HR 0.44; 95% CI 0.36–0.52) and stable disease was obtained by 60% and 33% of patients, respectively [11].

## Discussion

This exploratory analysis evaluated the safety and efficacy of cabozantinib in patients from CELESTIAL whose liver function deteriorated to Child–Pugh B status by Week 8 at the time of the first Child–Pugh investigator assessment. A majority of these patients had a Child–Pugh score of 6 at baseline, as determined by BCDM, whereas most of the patients who still had Child–Pugh A status at Week 8 had a Child–Pugh score of 5. Although investigator-assessed Child–Pugh grading and BCDM-determined Child–Pugh scoring were done independently, the majority of determinations were concordant. Cabozantinib appeared to have a manageable safety profile in the Child–Pugh B subgroup, with comparable rates to the overall cabozantinib group for dose reductions and discontinuations due to treatment-related AEs [11]. However, there were differences in the rates of some grade 3/4 AEs, including higher rates of fatigue, ascites, and thrombocytopenia in the Child–Pugh B subgroup compared with the overall cabozantinib group [11]. Higher rates of some grade 3/4 AEs were also noted in the placebo arm for the Child–Pugh B subgroup relative to the overall placebo group. As these grade 3/4 AEs occurred throughout the study, their incidence could be associated with reduced liver function, the course of the disease, or both. The higher incidence of thrombocytopenia with cabozantinib versus placebo in both the retrospective cohort and the overall study population and the absence of events in the placebo arm of the retrospective cohort could indicate an association with cabozantinib treatment. These data are consistent with the expected clinical manifestations of more advanced cirrhosis and portal hypertension [13–15], and suggest that patients with Child–Pugh B cirrhosis are at greater risk of experiencing treatment-emergent or treatment-related AEs compared with patients with Child–Pugh A cirrhosis, which represents nearly all of patients in the overall population.

Hazard ratios for OS and PFS indicate clinical benefit with cabozantinib in the Child–Pugh B subgroup. The outcomes with cabozantinib in patients with HCC and compromised liver function presented here are also supported by the outcomes of a CELESTIAL subgroup analysis based on baseline ALBI grades (an objective measure of liver function with higher grades associated with worse prognosis [16]) [17]. In the analysis by ALBI grade, a trend of improved OS and PFS with cabozantinib compared with placebo was observed irrespective of baseline grade [17]. It should be noted that a majority of patients in the Child–Pugh B subgroup had ALBI grade 2 cirrhosis at baseline.

The observed outcomes of cabozantinib in patients with reduced liver function should be interpreted with caution because of the retrospective nature of subgroup analyses and the relatively small size of the Child–Pugh B subgroup. As CELESTIAL did not allow for patients with Child–Pugh B status at study entry, we chose to analyse data from patients who developed Child–Pugh B cirrhosis on treatment. Further, 288/707 patients (41%) had unknown Child–Pugh status at Week 8. Prospective studies are required to further assess the efficacy and safety of cabozantinib in this patient population with Child–Pugh B status at start of therapy. A dose-escalation study in patients with HCC and Child–Pugh B cirrhosis will evaluate cabozantinib at three doses–20 mg, 40 mg, and 60 mg (NCT04497038) [18].

Previous retrospective and prospective studies have evaluated sorafenib, nivolumab, and regorafenib in patients with HCC and Child–Pugh B liver cirrhosis [19–24]. These studies focused on comparing Child Pugh A versus B; whereas this current study focused on outcomes in Child–Pugh B patients, as it was a retrospective subgroup analysis of a randomised study. In a prospective feasibility study of 300 patients with HCC treated with sorafenib, patients with Child–Pugh B cirrhosis had shorter PFS, time to progression, and OS than patients with Child–Pugh A status, with similar safety profiles [22]. In the GIDEON observational registry study of 3202 patients with HCC receiving sorafenib, including 666 patients with Child–Pugh B status, the incidence and type of AEs were consistent across Child–Pugh subgroups, with median overall survival longer for patients with Child–Pugh A versus B cirrhosis (13.6 vs. 5.2 months) [19]. In a study of 49 patients with Child–Pugh B cirrhosis receiving nivolumab from the CheckMate 040 study, treatment-related AEs associated with nivolumab resulting in treatment discontinuation were comparable with those for patients with Child–Pugh A cirrhosis, with a median OS in patients with Child–Pugh B status of 7.6 months [24]. In the REFINE observational study of patients with HCC, median OS with regorafenib was 16.0 months in patients with Child–Pugh A status compared with 8.0 months in patients with Child–Pugh B status [23]. In addition to these agents, cytotoxic anticancer agents have been evaluated in this patient population and have shown some level of efficacy and safety [25, 26].

Patients with Child–Pugh B cirrhosis and HCC have poor prognosis and considerable unmet medical need. The results presented in this retrospective analysis suggest encouraging safety and efficacy outcomes with cabozantinib in this patient population. Prospective studies involving cabozantinib are warranted in patients with advanced HCC and Child–Pugh B liver function.

## Supplementary Information

**Additional file 1.** 

## Data Availability

The datasets used and/or analysed during the current study are available from the corresponding author on reasonable request.

## Abbreviations

AE: Adverse event
ALBI: Albumin-bilirubin
ALT: Alanine aminotransferase
AST: Aspartate aminotransferase
BDCM: Biostatistics and Clinical Data Management
CI: Confidence interval
HBV: Hepatitis B virus
HCC: Hepatocellular carcinoma
HR: Hazard ratio
OS: Overall survival
PFS: Progression-free survival
