# Supplementary material for: Safety and efficacy of cabozantinib for patients with advanced hepatocellular carcinoma who advanced to Child–Pugh B liver function at study week 8: a retrospective analysis of the CELESTIAL randomised controlled trial
Source: BMC Cancer. 2022 Apr 9;22:377. doi: 10.1186/s12885-022-09453-z (PMC8994237; doi:10.1186/s12885-022-09453-z)
Supplement: Supplementary file 1 — Additional file 1. [file 12885_2022_9453_MOESM1_ESM.docx]

**Safety and efficacy of cabozantinib for patients with advanced hepatocellular carcinoma who advanced to Child-Pugh B liver function at study week 8: a retrospective analysis of the CELESTIAL randomised controlled trial.**

Anthony B. El-Khoueiry, Tim Meyer, Ann-Lii Cheng, Lorenza Rimassa, Suvajit Sen, Steven Milwee, Robin Kate Kelley, Ghassan K. Abou-Alfa

**List of ethics committee/independent review board approving the METEOR study protocol**

| Study Site | Name of Ethics Committee/Independent Review Board |
| --- | --- |
| **Australia** |  |
| The Royal Melbourne Hospital | Melbourne Health HREC, The Royal Melbourne Hospital |
| Ashford Cancer Centre Research | Bellberry Human Research Ethics Committee |
| Westmead Hospital | Melbourne Health HREC, The Royal Melbourne Hospital |
| St George Hospital | Melbourne Health HREC, The Royal Melbourne Hospital |
| St Vincent’s Hospital | Melbourne Health HREC, The Royal Melbourne Hospital |
| Royal Prince Alfred Hospital | Melbourne Health HREC, The Royal Melbourne Hospital |
| **Belgium** |  |
| University Hospital Antwerp | Ethics Committee University Hospital Antwerp |
| Centre Hospitalier de Jolimont | Ethics Committee University Hospital Antwerp |
| CHU of Liége | Ethics Committee University Hospital Antwerp |
| Universitair Ziekenhuis Gent | Ethics Committee University Hospital Antwerp |
| **Canada** |  |
| Princess Margaret Cancer Centre | University Health Network Research Ethics Board |
| Tom Baker Cancer Centre | Health Research Ethics Board of Alberta - Cancer Committee (HREBA-CC), Alberta Innovates - Health Solutions |
| **France** |  |
| Centre Hospitalier Universitaire Estaing | Comité de Protection des Personnes Ile-de-France III, Hôpital Tarnier |
| CHU de Bordeaux - Hôpital Haut-Lévéque | Comité de Protection des Personnes Ile-de-France III, Hôpital Tarnier |
| CHRU de Lille - Hôpital Claude Hurriez | Comité de Protection des Personnes Ile-de-France III, Hôpital Tarnier |
| Hôpital Henri Mondor | Comité de Protection des Personnes Ile-de-France III, Hôpital Tarnier |
| CHU de Nice- Hôpital de l’Archet II | Comité de Protection des Personnes Ile-de-France III, Hôpital Tarnier |
| Hôpital de la Croix Rousse | Comité de Protection des Personnes Ile-de-France III, Hôpital Tarnier |
| Centre Hospitalier Jean Minjoz | Comité de Protection des Personnes Ile-de-France III, Hôpital Tarnier |
| **Germany** |  |
| Universitätsklinikum Tübingen | Ethik-Kommission des Fachbereichs Medizin der Johann, Wolfgang Goethe-Universität |
| Charité, Campus Virchow Klinikum | Ethik-Kommission des Fachbereichs Medizin der Johann, Wolfgang Goethe-Universität |
| Klinikum rechts der Isar. TU Muenchen | Ethik-Kommission des Fachbereichs Medizin der Johann, Wolfgang Goethe-Universität |
| Klinikum Esslingen GmbH | Ethik-Kommission des Fachbereichs Medizin der Johann, Wolfgang Goethe-Universität |
| Universitätsklinikum Magdeburg A.ö.R | Ethik-Kommission des Fachbereichs Medizin der Johann, Wolfgang Goethe-Universität |
| Medizinische Klinik 1, Universitätsklinikum Frankfurt | Ethik-Kommission des Fachbereichs Medizin der Johann, Wolfgang Goethe-Universität |
| **Hong Kong** |  |
| Prince of Wales Hospital | Joint Chinese University of Hong Kong-New Territories East Cluster Clinical Research Ethics Committee, Prince of Wales Hospital |
| Queen Mary Hospital | Institutional Review Board of the University of Hong Kong/Hospital Authority Hong Kong West Cluster, Queen Mary Hospital |
| **Ireland** |  |
| Master Misericordae University Hospital | Clinical Research Ethics Committee of the Cork Teaching Hospitals |
| **Italy** |  |
| IRCCS Istituto Oncologico Veneto (IOV) | Comitato Etico Milano Area B-Ospedale Maggiore Poloclinico |
| Istituto Clinico Humanitas | Comitato Etico Milano Area B-Ospedale Maggiore Poloclinico |
| IRCCS Azienda Ospedaliero Universitaria San Martino | Comitato Etico Milano Area B-Ospedale Maggiore Poloclinico |
| Policlinico Universitario - Campus Biomedico | Comitato Etico Milano Area B-Ospedale Maggiore Poloclinico |
| P.O. di Faenza - Ospedale per gli Infermi | Comitato Etico Milano Area B-Ospedale Maggiore Poloclinico |
| U.O. Oncologia - Ospedale Infermi | Comitato Etico Milano Area B-Ospedale Maggiore Poloclinico |
| A.O.U. di Bologna - Policlinico S. Orsola Malpighi | Comitato Etico Milano Area B-Ospedale Maggiore Poloclinico |
| Fondazione IRCC Cá Granda Ospedale Maggiore Poloclinico | Comitato Etico Milano Area B-Ospedale Maggiore Poloclinico |
| A.O.U. di Padova | Comitato Etico Milano Area B-Ospedale Maggiore Poloclinico |
| Policlinico Universitario “A. Gemelli” | Segreteria Tecnico-Scientifica del Comitato Etico della Fondazione Policlinico Universitario A. Gemelli Universitá Cattolica del Sacro Cuore |
| **Korea, Republic of** |  |
| Severance Hospital, Yonsei University Health System | Yonsei University Health System, Severance Hospital, Institutional Review Board |
| Asan Medical Center | Asan Medical Center Institutional Review Board |
| Seoul National University Bundang Hospital | Seoul National University Bundang Hospital IRB |
| Seoul National University Hospital | Seoul National University Hospital Institutional Review Board |
| Samsung Medical Center | Samsung Medical Center Institutional Review Board |
| National Cancer Center | National Cancer Center IRB |
| Pusan National University Hospital | Pusan National University Hospital Institutional Review Board |
| **Netherlands** |  |
| LUMC | METC azM/UM |
| MUMC | METC azM/UM |
| AMC Amsterdam | METC azM/UM |
| **New Zealand** |  |
| Auckland District Health Board | Northern B Health and Disability Ethics Committee |
| **Poland** |  |
| ID Clinic | Komisja Bioetyczna przy Slaskiej Izbie Lekarskiej |
| **Romania** |  |
| Teo Health SA (“SF.Constantin” Hospital) | Academy of Medical Sciences - National Bioethics Committee for Medicines and Medical Devices |
| CF Cluj-Napoca Clinical Hospital | Academy of Medical Sciences - National Bioethics Committee for Medicines and Medical Devices |
| **Singapore** |  |
| Johns Hopkins Singapore International Medical Centre | NHG Domain Specific Review Board (DSRB) |
| National Cancer Centre Singapore | Singhealth Centralised Institutional Review Board (CIRB) |
| National University Hospital, NCIS | NHG Domain Specific Review Board (DSRB) |
| **Spain** |  |
| Hospital de Torrejón | CEIC Regional de la Comunidad de Madrid (CEIC-R) |
| Hospital Universitario Puerta de Hierro - Majadahonda | CEIC Regional de la Comunidad de Madrid (CEIC-R) |
| Hospital Universitario 12 de Octubre | CEIC Regional de la Comunidad de Madrid (CEIC-R) |
| Hospital General Universitario Gregorio Marañon | CEIC Regional de la Comunidad de Madrid (CEIC-R) |
| Hospital General Universitario Gregorio Marañon | CEIC Regional de la Comunidad de Madrid (CEIC-R) |
| **Taiwan** |  |
| National Taiwan University Hospital | Research Ethics Committee C National Taiwan University Hospital |
| National Cheng Kung University Hospital | Institutional Review Board National Cheng Kung University Hospital |
| Taipei Veterans General Hospital | Institutional Review Board of Taipei Veterans General Hospital |
| Chei Mei Hospital | Institutional Review Board of Chi Mei Medical Center |
| Taichung Veterans General Hospital | The Institutional Review Board of Taichung Veterans General Hospital |

| **Study Site** | **Name of Ethics Committee/Independent Review Board** |
| --- | --- |
| **Turkey** |  |
| Gaziantep Uni. Tip Fakultesi Onkoloji Hastanesi | Trakya Universitesi Tip Fakultesi Klinik Arastirmalar Etik Kurulu |
| Trakya Universitesi Tip Fakultesi Hastanesi, Iç Hastaliklari AD | Trakya Universitesi Tip Fakultesi Klinik Arastirmalar Etik Kurulu |
| **United Kingdom** |  |
| Christie NHS Foundation Trust, Christie Hospital | NRES Committee London-Harrow Bristol Research and Ethics Committee Centre |
| Medical Oncology, King’s College Hospital | NRES Committee London-Harrow Bristol Research and Ethics Committee Centre |
| Royal Free London NHS Foundation Trust | NRES Committee London-Harrow Bristol Research and Ethics Committee Centre |
| Clatterbridge Cancer Centre NHS Foundation Trust | NRES Committee London-Harrow Bristol Research and Ethics Committee Centre |
| University Hospitals Birmingham NHS Foundation Trust | NRES Committee London-Harrow Bristol Research and Ethics Committee Centre |
| **United States of America** |  |
| Memorial Sloan-Kettering Cancer Center | Institutional Review Board/Privacy Board, Memorial Sloan-Kettering Cancer Center |
| Columbia University Medical Center | Columbia University Medical Center IRB |
| Lahey Hospital & Medical Center | Lahey Clinic, Inc. Institutional Review Board |
| Westchester Medical Center Advanced Oncology and Infusion Center | New York Medical College Committee for Protection of Human Subjects |
| Beth Israel Deaconess Medical Center | Dana Farber/Harvard Cancer Center Office for Human Research Studies |
| Mayo Clinic | Mayo Clinic Institutional Review Board |
| University of Chicago | The University of Chicago Institutional Review Board |
| Kansas City Veterans Affairs Medical Center | Kansas City Veterans Affairs Medical Center IRB |
| Washington University School of Medicine | Washington University School of Medicine, Human Research Protection Office (HRPO) |
| Mary Crowley Cancer Research Centers - Medical City | Mary Crowley Cancer Research Center Institutional Review Board |
| UCSF Helen Diller Family Comprehensive Cancer Center | UCSF Institutional Review Board (Formerly CHR - Committee on Human Research), University of California |
| Seattle Cancer Care Alliance | Western Institutional Review Board |
| Comprehensive Cancer Centers of Nevada | Western Institutional Review Board |
| Swedish Cancer Institute | Western IRB |
| USC/Norris Comprehensive Cancer Center | University of Southern California Health Sciences Campus Institutional Review Board, LAC+USC Medical Center, General Hospital |
| Pacific Hematology Oncology Associates | Western Institutional Review Board (WIRB) |
| Medical Oncology Associates, PS | Western Institutional Review Board |
| University of Hawaii Cancer Center | Western Institutional Review Board |
| **Study Site** | **Name of Ethics Committee/Independent Review Board** |
| Piedmont Cancer Henderson, MD | Piedmont Healthcare Institutional Review Board |
| University of Florida | Western Institutional Review Board, Inc |

**Table A1 Change in liver function parameters in Child-Pugh B subgroup at Week 8 from baseline**

|  | **Child-Pugh B subgroup** | | **Overall population** | |
| --- | --- | --- | --- | --- |
|  | **Cabozantinib**  **(N = 51)** | **Placebo**  **(N = 22)** | **Cabozantinib**  **(N = 470)** | **Placebo**  **(N = 247)** |
| Albumin, mean (SD), g/L | n = 47 | n =21 | n = 341 | n = 173 |
| Baseline | 33.5 (3.4) | 34.3 (2.3) | 37.2 (4.3) | 37.9 (4.1) |
| Week 8 | 28.5 (3.7) | 29.3 (2.9) | 35.2 (5.6) | 37.0 (5.2) |
| Change from baseline | −5.0 (3.8) | −5.0 (2.4) | −2.0 (3.9) | −0.8 (3.6) |
| ALP, mean (SD), U/L | n= 47 | n = 21 | n = 344 | n = 176 |
| Baseline | 260.6 (189.7) | 281.8 (175.5) | 169.3 (123.4) | 161.1 (107.5) |
| Week 8 | 294.4 (400.6) | 389.1 (216.9) | 175.5 (183.9) | 201.0 (149.4) |
| Change from baseline | 33.7 (290.7) | 107.3 (157.9) | 6.1 (128.1) | 39.9 (90.1) |
| ALT, mean (SD), U/L | n= 47 | n = 21 | n = 337 | n = 173 |
| Baseline | 54.6 (33.2) | 62.2 (41.0) | 46.4 (35.1) | 44.5 (37.2) |
| Week 8 | 73. 3 (47.6) | 68.0 (48.5) | 70.3 (48.4) | 50.2 (52.3) |
| Change from baseline | 18.7 (32.5) | 5.9 (44.1) | 23.8 (39.9) | 5.7 (48.3) |
| AST, mean (SD), U/L | n= 46 | n = 21 | n = 334 | n = 172 |
| Baseline | 86.9 (42.3) | 90.3 (37.0) | 61.4 (38.9) | 55.8 (34.0) |
| Week 8 | 112.0 (66.7) | 140.7 (116.2) | 87.9 (60.4) | 73.9 (74.3) |
| Change from baseline | 25.1 (59.6) | 50.4 (98.6) | 26.4 (49.2) | 18.2 (63.8) |
| Bilirubin, mean (SD), μmol/L | n = 47 | n = 21 | n= 341 | n = 173 |
| Baseline | 16.7 (9.1) | 15.9 (9.3) | 12.4 (7.0) | 12.0 (6.2) |
| Week 8 | 34.8 (47.1) | 29.0 (20.2) | 17.5 (20.8) | 15.4 (11.8) |
| Change from baseline | 18.1 (45.6) | 13.1 (19.9) | 5.1 (18.8) | 3.4 (10.1) |
| Direct bilirubin, mean (SD), n, μmol/L | n= 44 | n = 21 | n = 326 | n= 170 |
| Baseline | 7.6 (4.9) | 7.2 (5.2) | 4.8 (3.3) | 4.5 (3.1) |
| Week 8 | 17.8 (33.4) | 16.4 (15.7) | 7.0 (13.7) | 6.5 (7.9) |
| Change from baseline | 10.1 (32.5) | 9.1 (15.2) | 2.2 (12.8) | 2.0 (7.1) |
| Indirect bilirubin, mean (SD), μmol/L | n = 44 | n = 21 | n = 326 | n = 170 |
| Baseline | 9.1 (4.6) | 8.7 (4.9) | 7.6 (4.1) | 7.6 (3.7) |
| Week 8 | 18.2 (17.7) | 12.7 (6.1) | 10.6 (9.0) | 8.9 (5.3) |
| Change from baseline | 9.0 (16.9) | 4.0 (5.8) | 3.0 (7.7) | 1.3 (4.2) |
| GGT, mean (SD), U/L | n = 47 | n = 21 | n = 344 | n= 176 |
| Baseline | 289.9 (277.4) | 388.3 (266.7) | 205.3 (219.6) | 212.9 (213.4) |
| Week 8 | 211.4 (301.0) | 427.5 (302.5) | 158.7 (205.6) | 247.3 (261.0) |
| Change from baseline | −78.4 (182.3) | 39.1 (232.9) | −46.3 (139.5) | 34.4 (157.0) |

ALP, alkaline phosphatase; ALT, alanine aminotransferase; AST, aspartate aminotransferase; GGT, gamma glutamyl transferase; SD, standard deviation
